# Effectiveness of topiramate for tobacco dependence in patients with depression; a randomised, controlled trial

**DOI:** 10.1186/1471-2296-9-28

**Published:** 2008-05-07

**Authors:** Javier García Campayo, Natalia Sobradiel, Marta Alda, Adoración Mas, Eva Andrés, Rosa Magallón, Arantxa Crucelaegui, Beatriz Sanz

**Affiliations:** 1 Servicio de Psiquiatría, Hospital Miguel Servet y Universidad de Zaragoza, Spain; 2 Servicio de Psiquiatría. Hospital de Alcañiz, Teruel, Spain; 3 Centro de Salud Perpetuo Socorro, Huesca, Spain; 4 Centro de Salud Arrabal, Zaragoza, Spain; 5 Servicio de Urgencias. Hospital de Tudela, Navarra, Spain; 6 Hospital de la Defensa, Zaragoza, Spain; 7 Grupo Aragonés de Investigación en Atención Primaria. Red de Actividades Preventivas y de Promoción de la Salud (REDIAPP) (G03/170). Instituto Aragonés de Ciencias de la Salud (IACS)

## Abstract

**Background:**

Tobacco dependence management is a multi-component intervention that includes pharmacological treatments such as Nicotine Substitution Therapy (NST) or bupropion, and psychological therapy. There are some preliminary reports on topiramate efficacy for tobacco dependence. The aim of this study is to determine whether topiramate is as effective as the standard NST treatment for tobacco cessation at 1-year follow-up in patients with depression.

**Method/design:**

*Design: *A randomised, controlled trial involving two groups, one of which is the control group consisting of patients on the standard pharmacological treatment for tobacco cessation (NST) and the other is the intervention group consisting of patients on topiramate as pharmacological treatment.

*Setting: *29 primary care health centres in the city of Zaragoza, Spain.

*Sample: *180 patients, aged 18–65 years, diagnosed with major depression, smoke more than 20 cigarettes/day, who have voluntarily asked for tobacco cessation therapy.

*Intervention: *A multi-component programme for tobacco cessation is offered to all of the patients in the study. This programme is made up of pharmacological therapy + group cognitive-behavioural therapy. Pharmacological therapy consists of NST for the control group and topiramate (200 mg/day) for the intervention group. Psychological therapy is made up of 16 sessions of manualised group therapy.

*Measurements: *Cessation will be assessed by patient self-declared abstinence, expired air carbon monoxide levels, and cotinine levels in saliva. Questionnaires on tobacco dependence, anxiety, depression, impulsiveness and self-efficacy will be administered. The interviewers will not know which group the patient belongs to (blind). The assessments will be carried out at baseline, D (cessation day) -1, D+1, weeks 1, 2, 3, 4, 6, 8, 10 and 13, and months 4, 5, 6, 8, 10 and 12.

*Main variables: *Tobacco cessation rates and tobacco dependence.

*Analysis: *The analysis will be per intent to treat. We will use the general linear models of the SPSS version 15 statistical package, to analyse the effect of the treatment on the result variable (tobacco cessation rate).

**Discussion:**

It is necessary to develop new and more effective pharmacological treatments for tobacco cessation. This randomised clinical trial will determine whether topiramate is effective for tobacco cessation in patients with depression.

**Trial registration:**

Current Controlled Trials ISRCTN93532081

## Background

Tobacco abuse is the main avoidable cause of death and disability in the world that is unrelated to others. The World Health Organization (WHO) estimates that 4 million persons died of tobacco-related disorders in 2002 [1]. In Spain, the latest available data from the National Health Survey (Encuesta Nacional de Salud) in 2003 [2] describe a prevalence of tobacco consumption of 31%, with a decrease in consumption by males, and a small but significant increase in consumption by females, mainly in adolescents and young women. For these reasons tobacco use is considered a public health problem and its treatment constitutes a challenge for health services.

At present, tobacco dependence management is carried out in a collaborative way in primary care and specialised settings by means of treatments that range from minimal to intensive intervention [3]. In the latter, a multi-component cessation intervention is applied by health professional specialised in tobacco dependence management [4,5]. The multi-component intervention includes first-line pharmacological treatments for tobacco such as Nicotine Substitution Therapy (NST) or bupropion, and psychological treatments. This intervention obtains a remission rate of 30–40% at 1-year follow-up [5,6]. As psychological therapy, cognitive-behavioural interventions, which can be administered on a group or individual basis, are used with follow-up sessions during the first year of abstinence. NST is administered by transdermal patches as basal treatment [7-9], added to nicotine lozenges [10] or nicotine chewing gums [11] that are self-administered occasionally depending on abstinence symptoms. Bupropion is a noradrenergic and dopaminergic antidepressant used for tobacco dependence because it acts on nicotinic receptors. It is twice as effective as a placebo for this treatment [12]. However, in psychiatric patients it should be used with caution owing to potential interactions with CYP2D6, an enzyme involved in the metabolisation of many psychiatric drugs [13].

Topiramate seems to be a new and promising pharmacological treatment for tobacco. A new antiepileptic drug, it was originally designed as an oral hypoglycaemic, and was subsequently approved as an anticonvulsant. It has increasingly been used in the treatment of numerous psychiatric conditions (binge eating disorders, bulimia nervosa, and alcohol dependence) and it has also been associated with weight loss, potentially relevant in reversing weight gain induced by psychotropic medications [14]. Topiramate has demonstrated its efficacy for the treatment of alcohol dependence in randomised controlled trials [15]. There are also some preliminary reports of its effectiveness for the treatment of tobacco dependence, despite these reports being substudies of other trials on alcohol dependence [16], uncontrolled studies with small samples [17] or short-term studies [18].

Recently, a group of smokers have been described as "special populations". These are groups with certain characteristics that require the need for more intensive treatments to attain tobacco cessation, owing to limitations in the use of pharmacological treatments (pregnancy) or because the cessation must be more immediate (patients with heart disorders or emphysema) [4]. One of these special populations is psychiatric patients in whom prevalence of tobacco dependence is higher than in the general population (52% vs. 31%) and effectiveness of cessation programmes are less effective [19]. In persons who smoke, there is a direct relationship between severity of psychiatric symptoms and tobacco dependence [20]. Specifically, patients with depression show more likelihood to become heavy smokers, to have more difficulties in stopping smoking, and to suffer from more abstinence symptoms than general population [21]. At present, there are not enough research studies on the effectiveness of topiramate for tobacco cessation and, specifically, in psychiatric populations such as patients with depression.

## Methods/Design

### Objectives

The general aim is to determine whether topiramate is at least as effective as NST in tobacco cessation at 1-year follow-up in patients with depression. The specific aims are to determine the factors that predict a good response to topiramate treatment and the possible differences in effectiveness in relation to gender.

### Design

**Figure 1:**
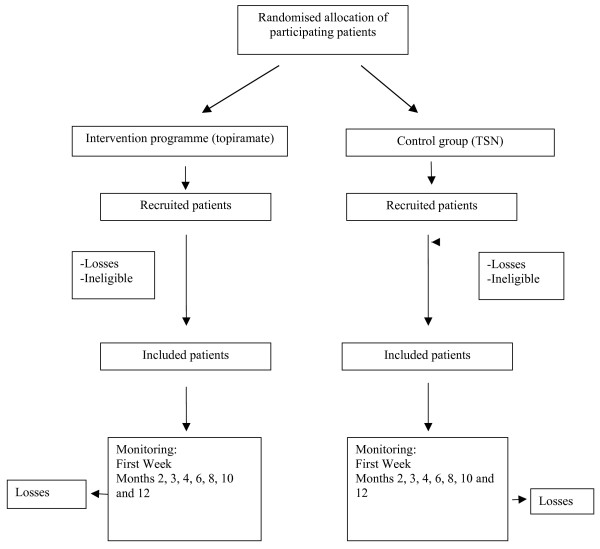
Flowchart: randomisation, sampling and monitoring of patients.

This is a controlled trial with a random allocation of patients into two alternative branches (see Figure 1):

1. Tobacco cessation standard treatment with NST (control group) and

2. Tobacco cessation treatment with topiramate (intervention group).

The evaluation of the treatment outcomes will be performed at patient level and they will be assessed individually.

### Setting and study sample

Patients will be recruited from any of the 29 primary health care centres in the city of Zaragoza (Aragonese Health Service), Spain. Patients will be recruited by doctors working in these primary care centres until the required sample is completed, without a quota of patients assigned for every centre.

Patients considered for **inclusion **are those aged 18–65 years, able to understand and read Spanish, who fulfil criteria for major depression (DSM-IV criteria), with scores on the Zung Self-Rating Depression Scale < 60 [22] (implying minimal to mild depression), who smoke more than 20 cigarettes/day, fulfil preparation state of change according to Prochaska & DiClemente [23], voluntarily ask for a tobacco cessation therapy, and sign informed consent. Those **excluded **will be patients with active psychosis and/or treatment with antipsychotic drugs, alcohol or drug abuse, and pregnancy or lactation.

### Randomisation, allocation and masking of study groups

Each patient will be allocated to either the intervention or the control group using a computer-generated random number sequence. The allocation will be carried out by an independent person, belonging to REDIAPP (Research Network on Preventative Activities and Health Promotion), who is not involved in the study. The method used to implement the random allocation sequence will be a central telephone. The sequence will be concealed until interventions are assigned. Patients agree to participate before the random allocation and without knowing which treatment they will be allocated to. Pharmacological treatment will be administered by a psychiatrist (JGC). Study personnel conducting psychological intervention and assessments (NS, AM, AC) will be masked to participants' treatment conditions.

### Intervention

#### Psychological intervention in both groups

A multi-component programme for tobacco cessation is offered to all of the patients in the study. This is made up of pharmacological therapy + group cognitive-behavioural therapy. The group is made up of 7–12 patients with depression and tobacco dependence, and is led by 2 therapists (a psychologist and a family doctor) trained in group therapy and tobacco cessation. Each session lasts 90 min., and the structure of every session and the contents are manualised and based on the standard programmes of this type [19]. It consists of 16 follow-up sessions: D (cessation day) -1, D +1, weeks 1, 2, 3, 4, 6, 8, 10 and 13, and months 4, 5, 6, 8, 10 and 12. Patients will be offered a phone number on which study personnel can solve problems and answer queries in relation to psychological (NS, AM) or pharmacological treatment (JGC).

#### Pharmacological intervention

##### Intervention group

In this group of patients, topiramate at doses used for the treatment of addictions (100–200 mg/day) will be administered [15-17].

##### Control group (standard treatment)

In this group, NST (nicotine patches) at usual doses (21 mg/day first and second fortnight, 14 mg/day third fortnight and 7 mg/day fourth and last fortnight), will be offered.

Pharmacological treatments will be financed by the grant. It will be administered by a psychiatrist (JGC). Duration of treatment, in both groups, will be 8 weeks. All patients will have free use of nicotine gums or lozenges during the two months of the treatment.

### Measurements

The study personnel that carried out the measurements (NS, AM, AC) will be unaware of which pharmacological treatment the patients is being administered ("blind"). The follow-up assessments will take place at baseline (clinical interview), D -1, D + 1, weeks 1, 2, 3, 4, 6, 8, 10 and 13, and months 4, 5, 6, 8, 10 and 12.

#### Variables and instruments of measurement (See Table 1)

**Table 1:** Study variables

**Instrument**	**Assessment area**	**Applied by**	**Time(s) of assessments**
Sampling form	Age, sex, inclusion/exclusion criteria	Family doctor	Baseline
Sociodemographic data form	Age, sex, marital status, educational level, Socio-economic group (28), occupation	Research assistant	Baseline
MINI psychiatric interview, depression and tobacco dependence modules (27)	Psychiatric diagnosis	Research psychiatrist	Baseline
Zung Self-Rating Depression Scale (22)	Severity of depression	Research assistant	Baseline and possible relapses
State of change (23)	State of change	Research assistant	Baseline
Self-declared abstinence	Tobacco abstinence	Research assistant	Baseline and all follow-up sessions*
Minnesota tobacco abstinence symptoms (24)	Tobacco abstinence	Research assistant	Baseline and all follow-up sessions*
Expired air carbon monoxide levels (25)	Tobacco abstinence	Research assistant	Baseline and all follow-up sessions*
Cotinine levels in saliva	Tobacco abstinence	Research assistant	Baseline and all follow-up sessions*
Fagerström test for Nicotine Dependence (29)	Tobacco dependence	Research assistant	Baseline and many follow-up sessions**
State-Trait Anxiety Inventory (STAI) (30)	Anxiety	Research assistant	Baseline and many follow-up sessions**
Plutchik Impulsivity Scale (31)	Impulsivity	Research assistant	Baseline and many follow-up sessions**
Visual analog scale	Efficacy self-perception	Research assistant	Baseline and many follow-up sessions**
Medical record	Pharmacological side-effect events	Research assistant	Baseline and many follow-up sessions**

*All follow-up sessions: D (cessation day) -1, D + 1, weeks 1, 2, 3, 4, 6, 8, 10 and 13, and months 4, 5, 6, 8, 10 and 12 (16 sessions in total) **Many follow-up sessions: weeks 1, 4, 8 and months 4, 6, 8, 10, 12.

##### Main outcome variables

In accordance with the aims of the study, the major outcome is tobacco cessation in patients with depression. The diagnosis of tobacco dependence will be made with the Spanish version of the MINI psychiatric interview substance dependence module adapted to tobacco [24]. This modification has been validated on a Spanish population with adequate psychometric properties [19]. Tobacco abstinence will be diagnosed by self-declared abstinence, self-administered Minnesota tobacco abstinence symptoms [25], expired air carbon monoxide [26], and cotinine in saliva. Tobacco abstinence, according to majoritarily-accepted criteria [27], has been defined using 2 concepts: 1. Occasional abstinence: In a follow-up visit, patients affirm that they have been abstinent, their levels of expired air carbon monoxide < 10 ppm, and cotinine levels in saliva < 5 ng/ml. Continuous abstinence: As of the visit at 30 days, patients affirm that they were abstinent the month before, expired air carbon monoxide levels < 10 ppm and cotinine levels in saliva < 5 ng/ml. Tobacco relapse will be diagnosed with the MINI psychiatric interview substance dependence module adapted to tobacco [24].

The diagnosis of depressive disorder will be made with the Spanish version of the MINI psychiatric interview, depression module [24], and the severity of the depression with the Spanish version of the Zung Self-Rating Depression Scale [22]. Recruitment will only include patients with depressive disorder and Zung scale scores < 60, which implies minimal to mild depression [22]. MINI psychiatric interview depression module [24], will be also used as criteria for depression relapse.

##### Secondary variables

-The following socio-demographic data will be collected: sex, age, marital status (single, married/relationship, separated/divorced, and widowed), education (no studies, primary, lower secondary, upper secondary, university), occupation and social class (I, II, IIIN, IIIM, IV and V of the British Registrar General's Scale) [28].

-Tobacco dependence as measured by the Spanish version of the Fagerström test for Nicotine Dependence [29].

-Anxiety trait and state as measured by the Spanish version of the State-Trait Anxiety Inventory (STAI) [30].

-Impulsivity as measured by the Spanish version of the Plutchik Impulsivity Scale [31].

-Visual analogue scale for efficacy self-perception (range 0–10).

-Pharmacological side-effect events from the medical record.

### Statistical methods

#### Sample size

To calculate the sample size we consider the tobacco cessation rate at 1 year follow-up as the main outcome variable. On the basis of published research data [5,6], we assume that this will be 35% in the control (NST) group, and we aim to detect a difference of 25% or more between any of the two groups (control and intervention). Published studies place placebo response at 10% [32]. Accepting an alpha risk of 0.05 and a beta risk of < 0.20 in a bilateral contrast, we would need 90 patients in each group [33].

#### Analysis strategy

The analysis will be per intent to treat. First we will compare the intervention group with the control group in order to verify that there are no significant differences between the two groups (socio-demographic characteristics, clinical baseline data, etc). We will use the mean (standard deviation) in the continuous variables and percentages in the categorical variables. For comparisons we will use the Student-t test for continuous variables and the Chi-squared test for categorical variables. Non-parametric tests may also be used.

The main variables of the result are the percentage of patients with tobacco cessation (patient's self-declared abstinence, expired air carbon monoxide levels, self-administered Minnesota tobacco abstinence symptoms, and cotinine levels in saliva), and tobacco dependence (Fagerström test for Nicotine Dependence scoring) at 1-year.

Process variables include severity of the depression (Zung Self-Rating Depression Scale), anxiety trait and state (STAI), impulsivity (Plutchik Impulsivity Scale), and efficacy self-perception (visual analogue scale).

We will use the general linear models of the SPSS 15 statistical package, to analyse the effect of the treatment on the categorical result variables (tobacco cessation rate). We will use the analyses of linear mixed models to analyse the effect of the continuous process variables (depression, anxiety, impulsivity and efficacy).

### Ethical aspects

Informed consent will be obtained from the participants before they are aware of which group they are to be included in. Before they give their consent, the patients will be provided with a general overview of the aims and characteristics of the study and the multi-component intervention. They will also be informed that they will be participating voluntarily, and that they can choose to withdraw at any time with the guarantee that they will continue to receive the treatment considered most appropriate by their doctor.

The study follows Helsinki Convention norms and posterior modifications and the Declaration of Madrid of the World Psychiatric Association. The Study Protocol was approved by the Ethical Review Board of the regional health authority in February 2007 (ref: FIS PI06/1462).

### Forecast execution dates

Initial recruitment of patients: March 2008

Finalisation of patient recruitment: December 2008

Finalisation of patient monitoring period: December 2009

Publication of results: June 2010

## Discussion

Effectiveness of multi-component programmes with standard pharmacological treatments (NST) for tobacco cessation in the general population is still unsatisfactory, with cessation rates at 1-year follow-up ranging from 30–40% [5,6]. These rates are even less successful in special populations such as psychiatric patients [19]. For these reasons, it seems necessary to develop new and more effective pharmacological treatments. One of the most promising treatments is topiramate because it has demonstrated effectiveness on tobacco cessation in preliminary studies [16-18], and it is useful in many psychiatric conditions [14].

The strength of the study is that, to our knowledge, this is the first randomised, controlled trial of topiramate for tobacco cessation, with the standard treatment (NST) as comparator. The psychological intervention of the multi-component programme is the usual group cognitive-behavioural therapy used in these programmes [19]. In addition, the modulator role of some important variables (such as anxiety, impulsivity, state of change, self-efficacy) in patients with depression has also been assessed.

A number of potential limitations may be difficulties in recruitment, due to lack of motivation for tobacco cessation in psychiatric patients, and the low cessation rate in this population making it difficult to interpret the results. The concept of states of change has also been criticised, so its utility may be doubtful.

## Competing interests

The authors declare that they have no competing interests.

## Authors' contributions

JGC is the principal researcher and developed the original idea for the study. The study design was further developed by MA, NS and RM. NS, AM, AC and BS participated in the design and planning of the intervention that is evaluated here. EA developed the statistical methods. All authors have read and corrected draft versions and approved the final version.

## Pre-publication history

The pre-publication history for this paper can be accessed here:


